# Breast Adenoid Cystic Carcinoma: Recognizing a Rare Pathological Diagnosis

**DOI:** 10.7759/cureus.101447

**Published:** 2026-01-13

**Authors:** Prajakta Attarde, Shital Parekh

**Affiliations:** 1 Histopathology, Milton Keynes University Hospital, Milton Keynes, GBR

**Keywords:** adenoid cystic carcinoma (acc), breast acc, cd117 (c-kit), myb-fib fusion, triple-negative breast cancer (tnbc)

## Abstract

Adenoid cystic carcinoma (ACC) of the breast is an exceedingly rare neoplasm. It exhibits a distinct dual cell population and a favorable prognosis despite its often triple-negative immunophenotype. We present a rare case of ACC diagnosed on core needle biopsy and confirmed in the resection specimen. The histopathological features, immunohistochemical profile, and relevant literature are discussed. This case emphasizes the importance of recognizing this rare entity when encountered in breast biopsies and the necessity for a collaborative, multidisciplinary approach to management.

## Introduction

Adenoid cystic carcinoma (ACC) of the breast is an uncommon type of breast cancer, representing fewer than 0.1% of all cases. Reported relative survival rates are high, with 98.1% at five years, 94.9% at 10 years, and 91.4% at 15 years [1]. ACC primarily affects women, with male cases being extremely uncommon. Histologically, it consists of a combination of tubular-trabecular, cribriform, and solid growth patterns, which can lead to frequent clinical misdiagnoses. This cancer is considered a distinct subtype of triple-negative breast cancer (TNBC), characterized by low Ki-67 expression, a generally favorable prognosis, and infrequent axillary lymph node involvement. However, local recurrence and distant metastasis, most commonly to the lungs, can occur within a decade of diagnosis. Given its complex histopathology, a thorough clinical and pathological evaluation is essential for accurate diagnosis and management [1]. ACCs of the breast have favorable outcomes in comparison with other types of breast cancer and ACCs that occur in salivary glands [2]. In this report, we present a case of ACC of the breast initially diagnosed on core biopsy and subsequently confirmed on resection specimen.

## Case presentation

An elderly woman presented to the breast unit with a history of a lump in her right breast. She is a postmenopausal lady, never took hormone replacement therapy, is a mother of two and breastfed them, and has no other history of breast diseases. Clinical examination revealed an irregular lump behind the right nipple extending to the outer quadrant. Ultrasound confirmed a 35 mm lobulated mass with a solid cystic appearance. Given the clinical and imaging findings, a core needle biopsy of the lesion was performed.

Biopsy findings

**Figure 1:**
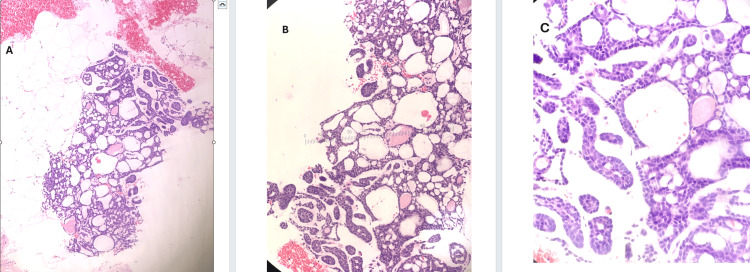
Core biopsy showing cribriform tumor with intraluminal basement membrane-like material (H&E: A, 10×; B, 20×; C, 40×)

Microscopically, it showed a grade 2 invasive carcinoma with a cribriform growth pattern (adenoid cystic carcinoma) (Figure 1; source: pathology department, Milton Keynes University Hospital {MKUH}).

Immunohistochemical analysis showed strong and diffuse positivity for cluster of differentiation 117 (CD117) (c-KIT) in the tumor cells. Myoepithelial cells, identified by p63 and smooth muscle myosin (SMM) staining, were present surrounding the tumor nests, confirming the biphasic nature characteristic of ACC. Tumor cells also demonstrated positive staining for epithelial cadherin (E-cadherin) and cytokeratin 5 (CK5). The tumor was negative for estrogen receptor (ER) (0/8), progesterone receptor (PR) (0/8), and human epidermal growth factor receptor 2 (HER2) (score of 0, non-amplified). The overall features were consistent with a diagnosis of grade 2 adenoid cystic carcinoma of the breast. The core biopsy was categorized as B5b according to the UK National Health Service Breast Screening Programme (NHSBSP) guidelines, indicating a malignant lesion requiring surgical excision. The case was also discussed in the multidisciplinary meeting.

Immunohistochemistry of the core biopsies

**Figure 2:**
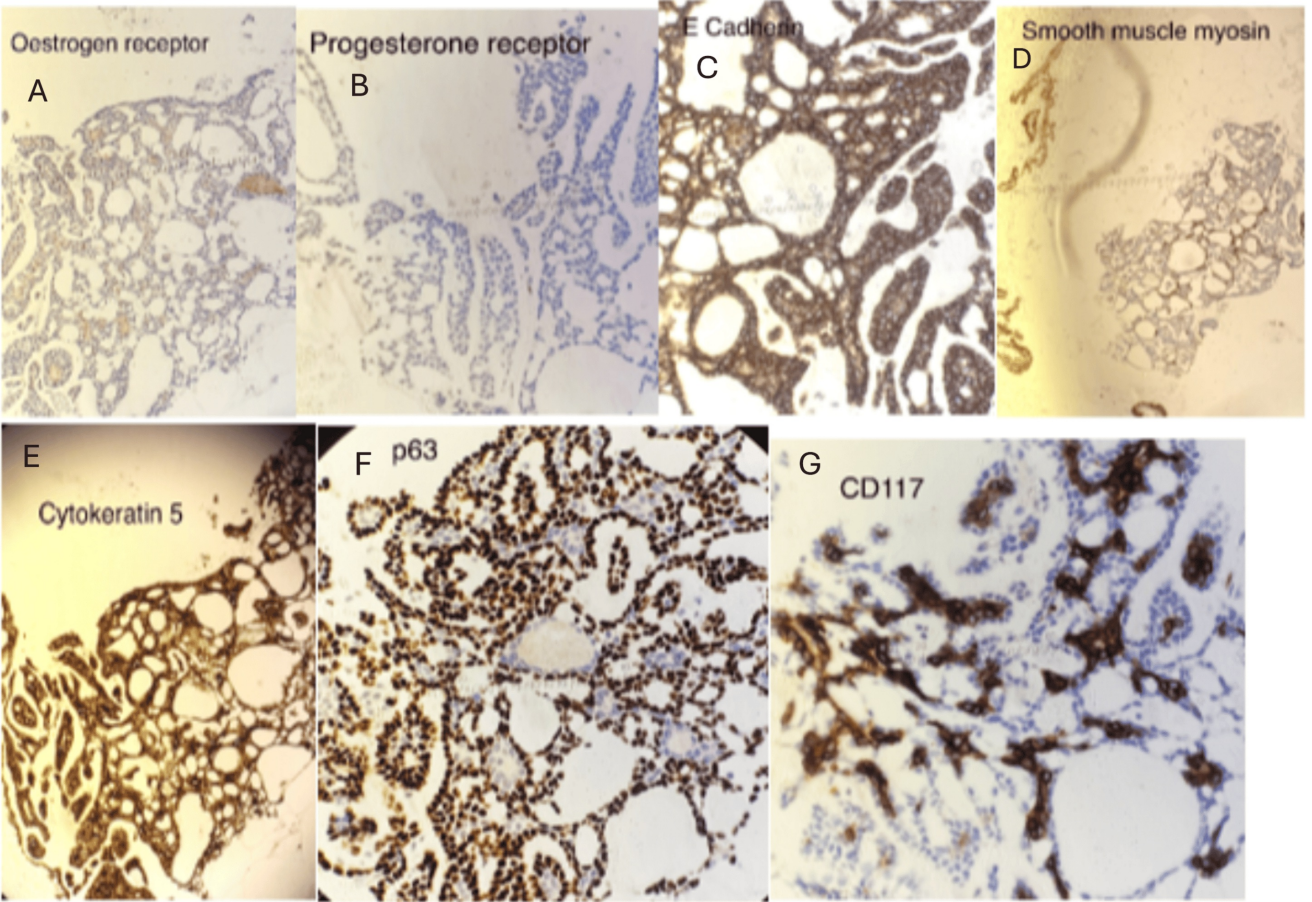
IHC profile of the tumor: A, estrogen receptor; B, progesterone receptor; C, E-cadherin; D, smooth muscle actin; E, cytokeratin 5; F, p63; and G, CD117 IHC, immunohistochemistry; E-cadherin; epithelial cadherin; CD117, cluster of differentiation 117

The tumor cells are positive for CD117, p63, E-cadherin, CK5, and SMM. ER is 0/8, and PR is 0/8 (Figure 2; source: pathology department, MKUH).

Surgical management

The patient underwent a mastectomy and sentinel node biopsy.

**Figure 3:**
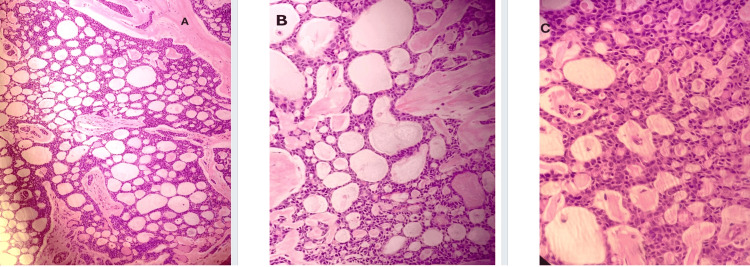
Microscopy images of the tumor from the mastectomy specimen (H&E: A, 10×; B, 20×; C, 40×), confirming the findings

The mastectomy specimen revealed a tumor measuring 30 mm in greatest dimension. All surgical margins were clear. Lymph node assessment showed no metastatic involvement in the sampled nodes (one sentinel lymph node and one intramammary lymph node). Histologically, the tumor was graded as grade 2 (Figure 3; source: pathology department, MKUH). Perineural invasion was observed, while lymphovascular invasion was not identified. No in situ carcinoma was present. The Nottingham prognostic index (NPI) was calculated at 3.6.

## Discussion

Primary adenoid cystic carcinoma of the breast represents a rare histological entity within mammary malignancies. Its occurrence at this site is notable, as ACC more commonly arises in the salivary glands and other head and neck regions. The diagnosis on core needle biopsy can be particularly challenging due to the limited tissue sample and the need to differentiate it from more common breast lesions [3].

The characteristic histopathological features, including the presence of cribriform, tubular, and/or solid patterns with a distinct basement membrane material surrounding the epithelial islands, are crucial for diagnosis. Immunohistochemical staining for markers such as c-KIT (CD117) and myoepithelial markers (p63 and smooth muscle actin) aids in confirming the diagnosis and excluding mimics. The classic subtype, which represents the majority of cases, generally shows an excellent prognosis with infrequent regional or distant metastases, resulting in better overall survival compared to invasive breast carcinoma. Histologically, it displays cribriform patterns within tubular structures composed of both epithelial and myoepithelial cells, with glandular spaces containing mucin. This form of ACC typically lacks marked nuclear atypia or necrosis and exhibits low mitotic activity. On immunohistochemistry, epithelial cells stain positively for CK7, CK8, and epithelial membrane antigen (EMA), while myoepithelial cells are positive for CK5/6 and p63. The luminal component shows strong CD117 positivity. Accurate diagnosis depends on correlating histological appearance with immunostaining results. For example, the tubular areas of classic ACC can be differentiated from microglandular adenosis and tubular carcinoma based on cellular makeup and mucin production, whereas distinguishing its cribriform regions from collagenous spherulosis and cribriform carcinoma is equally important.

In the cribriform component, adenoid cystic carcinoma and in situ or infiltrating cribriform carcinoma both present as breast masses, while collagenous spherulosis does not form a mass. All three have an epithelial component, but the myoepithelial component is always present in adenoid cystic carcinoma, may be present or absent in cribriform carcinoma, and is absent in collagenous spherulosis. Spaces containing mucin and basement membrane material are seen in all three. Hormone receptors (estrogen and progesterone) are negative in adenoid cystic carcinoma but positive in both cribriform carcinoma and collagenous spherulosis. CD117 immunostaining is positive only in adenoid cystic carcinoma and negative in the other two.

In the tubular component, adenoid cystic carcinoma, tubular carcinoma, and microglandular adenosis all have epithelial components. Myoepithelial cells are present only in adenoid cystic carcinoma and absent in the other two. Spaces containing mucin and basement membrane material are present in adenoid cystic carcinoma but absent in tubular carcinoma and microglandular adenosis, though the latter lacks eosinophilic material. Hormone receptors are negative in adenoid cystic carcinoma and microglandular adenosis but positive in tubular carcinoma. The solid-basaloid subtype is characterized by compact nests of basaloid cells showing prominent nuclear atypia, frequent mitotic figures, and areas of necrosis. Perineural invasion is often observed. The differential diagnosis includes other basaloid-type carcinomas and small-cell neuroendocrine carcinoma, though the presence of typical adenoid cystic carcinoma (ACC) patterns supports the diagnosis. This variant tends to behave more aggressively, frequently exhibiting axillary lymph node involvement, perineural spread, and a higher risk of local recurrence and distant metastasis, most commonly to the lungs, bones, and skin [3,4].

A characteristic t(6;9) genetic translocation produces a *MYB-FIB* fusion, leading to the activation of the *MYB* oncogene; this is particularly well-documented in the more common salivary gland tumors, where the fusion frequently occurs. Metastasis to axillary lymph nodes is exceedingly uncommon, though some patients may experience local recurrence or develop pulmonary metastases, sometimes occurring long after the initial diagnosis or treatment. Overall, the prognosis for this tumor group is generally favorable. The connection between microscopic grading and clinical outcome is still debated, with some studies suggesting that high-grade or anaplastic-appearing tumors could follow a more aggressive course [5].

Management typically involves surgical excision with adequate margins. The role of adjuvant therapies, such as radiotherapy and systemic therapy, is less defined due to the rarity of the disease, and treatment decisions should be individualized based on tumor characteristics and clinical context [6]. Long-term follow-up is essential to monitor for local recurrence and the potential for late distant metastasis [7].

Prognosis is generally favorable with low recurrence and rare distant metastasis.

## Conclusions

This case emphasizes the importance of recognizing the histopathological and immunohistochemical hallmarks of ACC of the breast. Accurate diagnosis, even on core biopsy, can guide appropriate surgical management. Due to its rarity, the awareness of its clinical course and differential diagnosis is essential for pathologists and oncologists.

Primary adenoid cystic carcinoma (ACC) of the breast is a rare cancer that requires careful histopathological evaluation for diagnosis, especially on core needle biopsy. It is defined by a cribriform growth pattern and biphasic cell population, with immunohistochemical markers such as CD117, p63, and SMM essential for confirming the diagnosis and ruling out other entities. Breast ACC usually shows a triple-negative profile (ER-, PR-, and HER2-). Treatment is mainly surgical, with adjuvant therapy considered individually. Due to the risk of late metastasis, long-term follow-up is necessary.
